# Blocking of EGFR Signaling Is a Latent Strategy for the Improvement of Prognosis of HPV-Induced Cancer

**DOI:** 10.3389/fonc.2021.633794

**Published:** 2021-09-27

**Authors:** Jianfa Qiu, Feifei Hu, Tingting Shao, Yuqiang Guo, Zongmao Dai, Huanhuan Nie, Oluwatayo Israel Olasunkanmi, Yue Qi, Yang Chen, Lexun Lin, Wenran Zhao, Zhaohua Zhong, Yan Wang

**Affiliations:** ^1^ Department of Microbiology, Harbin Medical University, Harbin, China; ^2^ Department of Obstetrics, the First Affiliated Hospital of Harbin Medical University, Harbin, China; ^3^ College of Bioinformatics Science and Technology, Harbin Medical University, Harbin, China; ^4^ Department of Cell Biology, Harbin Medical University, Harbin, China

**Keywords:** EGFR, hNSC, head and neck squamous cell carcinoma, cesC, human papillomavirus, E7

## Abstract

Human papillomavirus (HPV) is a double-stranded DNA (dsDNA) virus, and its high-risk subtypes increase cancer risks. However, the mechanism of HPV infection and pathogenesis still remain unclear. Therefore, understanding the molecular mechanisms and the pathogenesis of HPV are crucial in the prevention of HPV-related cancers. In this study, we analyzed cervix squamous cell carcinoma (CESC) and head and neck carcinoma (HNSC) combined data to investigate various HPV-induced cancer common features. We showed that epidermal growth factor receptor (EGFR) was downregulated in HPV-positive (HPV+) cancer, and that HPV+ cancer patients exhibited better prognosis than HPV-negative (HPV−) cancer patients. Our study also showed that TP53 mutation rate is lower in HPV+ cancer than in HPV− cancer and that TP53 can be modulated by HPV E7 protein. However, there was no significant difference in the expression of wildtype TP53 in both groups. Subsequently, we constructed HPV-human interaction network and found that EGFR is a critical factor. From the network, we also noticed that EGFR is regulated by HPV E7 protein and hsa-miR-944. Moreover, while phosphorylated EGFR is associated with a worse prognosis, EGFR total express level is not significantly correlated with prognosis. This indicates that EGFR activation will induce a worse outcome in HPV+ cancer patients. Further enrichment analysis showed that EGFR downstream pathway and cancer relative pathway are diversely activated in HPV+ cancer and HPV− cancer. In summary, HPV E7 protein downregulates EGFR that downregulates phosphorylated EGFR and inhibit EGFR-related pathways which in turn and consequently induce better prognosis.

## 1 Introduction

Tumor can be caused by several factors (1). A virus is a small pathogen that often causes pathological changes or diseases in the target host (2). Some viral infections have been linked to be essential factors that induce numerous forms of cancer such as liver cancer and nasopharyngeal carcinoma (3). Virus lifecycle requires intracellular environment owing to its simple structure (4). It hijacks the cell’s complex protein and nucleic acid synthesis system for self-proliferation and also controls the functional protein of cells to modulate the normal cell signaling pathway (5).

Human papillomavirus (HPV) is a nonenveloped double-stranded DNA (dsDNA) tumor virus. Almost all cervical squamous cell carcinoma and about 40% of head and neck cancers are consequences of HPV infection (6, 7). HPV preferably infect the mucosal layer, and no evidence shows that HPV has the ability to infect other cells except basal cells of the epithelia. Basal cell is the high differential ability cell of the epithelia. Hence, host cell development and differentiation ability are probably required for HPV infection (8). The carcinogenesis of squamous cell carcinoma is often accompanied by changes in development-related functions (9). Therefore, epithelia development-regulated protein may be the key target of HPV infection and oncogenesis. HPV genome encodes seven early-phase proteins (E1 to E7) and two late-phase proteins (L1 and L2) for its proliferation. E6 and E7 proteins can modulate p53 and Rb through downregulation or inhibition, which is the basic mechanism of HPV+ cancer genesis (7). Therefore, E6 and E7 can be regarded as the most essential HPV oncogenic proteins (10). Due to its small genome and limited virus-encoded protein, virus proteins require high efficiency and multifunctionality for complicated manipulation. For example, evidences showed that E6 and E7 proteins can interact with many human proteins and participate in a lot of biological processes (11). Likewise, HPV capsid protein L1 and L2 have been reported to interact with human proteins (12).

Many studies have examined HPV infection features in several types of cancers and HPV infection induced cancers have also been sufficiently investigated. For example, over 90% of the occurrence of cervix squamous cell carcinoma (CESC) is attributed to HPV infection (13). Also, head and neck carcinoma (HNSC) highly linked to HPV infection (14). Epidermal growth factor receptor (EGFR) is a cancer-related gene and it also has been reported to be a potential biomarker of HPV infection (15–17). There are also studies that have demonstrated that some subtypes of HNSC exhibit higher HPV infection rate than other subtypes, and that HPV copy number is lower in HPV infected subtypes (18). Furthermore, EGFR is associated with HPV-related cancer prognosis. It was reported that EGFR and pEGFR (phosphorylated epidermal growth factor receptor) are potential biomarkers of prognosis for oropharyngeal cancer (19). In cervical cancer, EGFR signaling can be affected by Hippo/YAP pathway and eventually influence cancer progression (20). Some reports suggested that EGFR expression can be regulated by HPV E5 protein (17), while contradicting reports showed that E5 protein does not regulate the expression of EGFR and cancer prognosis (21). Other reports showed that EGFR can be possibly regulated by miRNA. For example, in HPV-infected patients, smoking-induced control of miR-133a-3p regulates the expression of EGFR and human antigen R (HuR) (22). Hence, EGFR expression in HPV-infected cancer may be regulated by multiple factors such as existing complex mechanisms and HPV viral protein. However, no study has completely established the HPV protein is the key modulator of EGFR.

Since most of the previous studies focused on comparing single cancer type or HPV+ groups with normal group, there are limited studies that focused on multiple-cancer types or compared HPV+ cancer with HPV− cancer. Therefore, it is noteworthy to investigate EGFR regulating mechanisms in a multiple-cancer type. Owing to existing EGFR-targeted drugs, EGFR would be a potential target for HPV-induced cancer prognosis improvement.

Our study analyzed CESC and HNSC combined data at multiple levels including mRNA, miRNA, SNV, and protein expression level. We also constructed a global network with HPV proteins, HPV differentially expressed genes, and miRNAs in HPV+ cancers versus HPV− cancers. Through the network, our study showed that EGFR is regulated by HPV E7 protein and downregulated by miR-944. Furthermore, our findings showed that pEGFR and its up- and downstream protein activation are negatively correlated with HPV+ cancer survival. These findings are evidences that EGFR is regulated in a complex mechanism and that E7 is the HPV protein that regulates EGFR expression in HPV-induced cancer.

## 2 Results

### 2.1 HPV-Positive Cancer Patients Are Significantly Different From HPV-Negative Cancer Patients in Gene Expression and They Show Higher Survival Possibility

**Figure 1:**
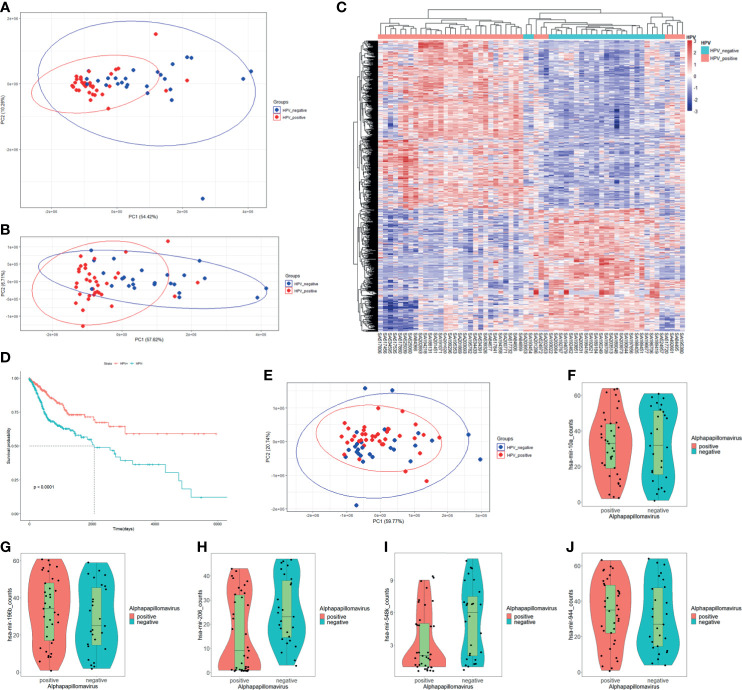
mRNA expression, miRNA expression, and prognosis differences in HPV+ cancers against HPV− cancers. **(A)** Principal component analysis (PCA) plot of the RNA-Seq data of CESC and HNSC samples within PCAWG program of ICGC database. HPV+ samples are marked in red; HPV− samples are marked in blue. **(B)** Redrawn PCA plot after outlier was removed. HPV+ samples are marked in red, and HPV− samples are marked in blue. **(C)** Differentially expressed genes (DEGs) heatmap of HPV+ group vs. HPV− group. Gene FPKMs were scaled with z-score by samples. Pearson’s correlation coefficients were taken for samples and gene clustering. **(D)** KM-plot of HPV+ patients and HPV− patient survival status from TCGA CESC and HNSC project. **(E)** PCA plot of miRNA-Seq data of CESC and HNSC sample within the PCAWG program of ICGC database. HPV+ samples are marked in red, HPV− samples are marked in blue. **(F–J)** hsa-miR-10a, hsa-miR-196b, hsa-miR-206, hsa-miR-548k, and hsa-miR-944 expression status in HPV+ samples and HPV− samples respectively.

In order to understand the relationships between HPV regulation and cancer, we selected the two most common HPV-related cancers which are CESC and HNSC for combined analysis. For RNA-Seq read counts matrix from ICGC database, principal component analysis (PCA) showed sample distribution of HPV+ and HPV− samples (**Figure 1A**). After removing the outlier at the lower right area, PCA plot was redrawn as displayed in **Figure 1B**, in which, HPV+ samples showed different distribution patterns against HPV− samples. The different distribution pattern shows that HPV+ cancer is distinct from HPV− cancer in gene expression. Further differentially expressed gene analysis was carried out by grouping samples by their HPV infection status. Eight hundred thirty-four differentially expressed genes (DEGs) were screened, and these genes basically distinguished HPV+ from HPV− samples (**Figure 1C**). Survival analysis based on clinical data from FireBrowse database showed that HPV+ patients had better prognosis compared with HPV− patients (**Figure 1D**). It implies that different survival rates are attributed to DEGs to some degree.

The miRNA-Seq read counts data were also analyzed using PCA and differential expression analysis. PCA distribution showed no obvious difference between HPV+ and HPV− samples (**Figure 1E**), which indicates that there is no clear difference in miRNA expression level between HPV+ cancer and HPV− cancer. Differentially express analysis further showed that only five miRNAs were significantly differentially expressed. They were hsa-miR-944, hsa-miR-196, hsa-miR-206, hsa-miR-10a, and hsa-miR-548k (**Figures 1F–J**). Further screening of the target in intersection of differentially expressed miRNAs showed only hsa-miR-944, hsa-miR-206, and hsa-miR-548k DEG targets, which signifies that hsa-miR-196 and hsa-miR-10a probably do not participate in DEG-related functions although they were differentially expressed. Both hsa-miR-196 and hsa-miR-10a were upregulated in HPV+ cancers. The differential expression may be related to HPV proliferation. Small miRNA expressing differences between HPV+ cancer and HPV− cancer shows that only few miRNAs participate in HPV infection-specific regulation and most of them are only cancer related or are steadily expressed in both situations.

### 2.2 TP53 Mutation Proportion Is Lower in HPV+ Cancer Than in HPV− Cancer

**Figure 2:**
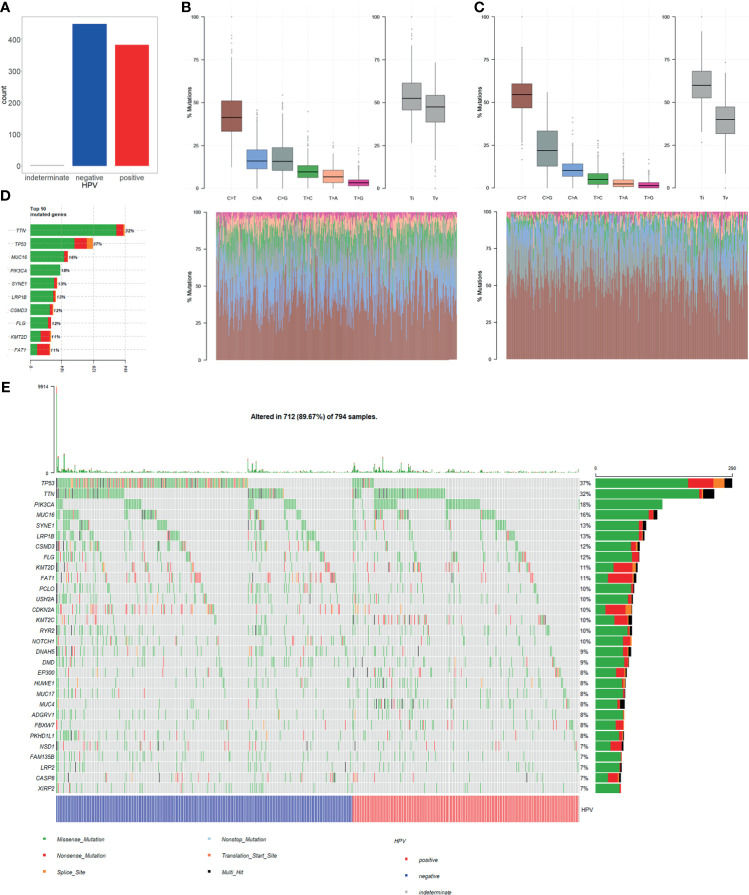
Single nucleotide variation (SNV) in HPV+ cancers and HPV− cancers. **(A)** Total numbers of HPV+ samples and HPV− samples in SNV data of TCGA CESC and HNSC project. MUSE software processed SNV data was used in our study. **(B)** HPV+ samples single nucleotide mutation-type proportions. **(C)** HPV− samples single nucleotide mutation-type proportions. **(D)** Top 10 highly mutation rates and mutated sample counts of mutated genes. **(E)** Oncoplot of the top 30 highly mutated genes; samples were grouped by HPV infection status.

Single nucleotide variation (SNV) analysis was done for CESC and HNSC combined data. Comparing HPV+ and HPV− groups, the number of samples was almost the same (**Figure 2A**). In determining the nucleotide variation type, we showed that HPV+ sample variation types and rates differ from that of HPV− samples. It showed that C>G mutation rates of HPV+ samples are higher when compared with C>A mutation, while they are almost the same in HPV− samples (**Figures 2B, C** **)**. At gene level for all samples, TP53 ranked at the 2nd place for single nucleotide mutation for all the genes. Moreover, TP53 mutation takes up 37% samples of the total mutation and it got the first place of all matched genes (**Figure 2D**). These results suggest that TP53 mutation plays a critical role in CESC and HNSC.

Furthermore, we selected top 30 genes with the highest mutation frequency and used oncoplot to show their mutation rates in each of the samples. The result showed that the number of TP53 mutation is significantly higher in HPV− cancer than in HPV+ cancer. Whereas most of the genes with high mutation rates showed no significant difference in both groups (**Figure 2E**). This result suggests that TP53 mutation is an important mechanism for the occurrence of HPV−. However, HPV+ cancer shows no relative involvement with TP53 mutation, and cancer occurrence may be involved in other mechanisms.

### 2.3 HPV Protein Regulation of Human Protein Is an Important Mechanism for the Occurrence of HPV+ Cancer

**Figure 3:**
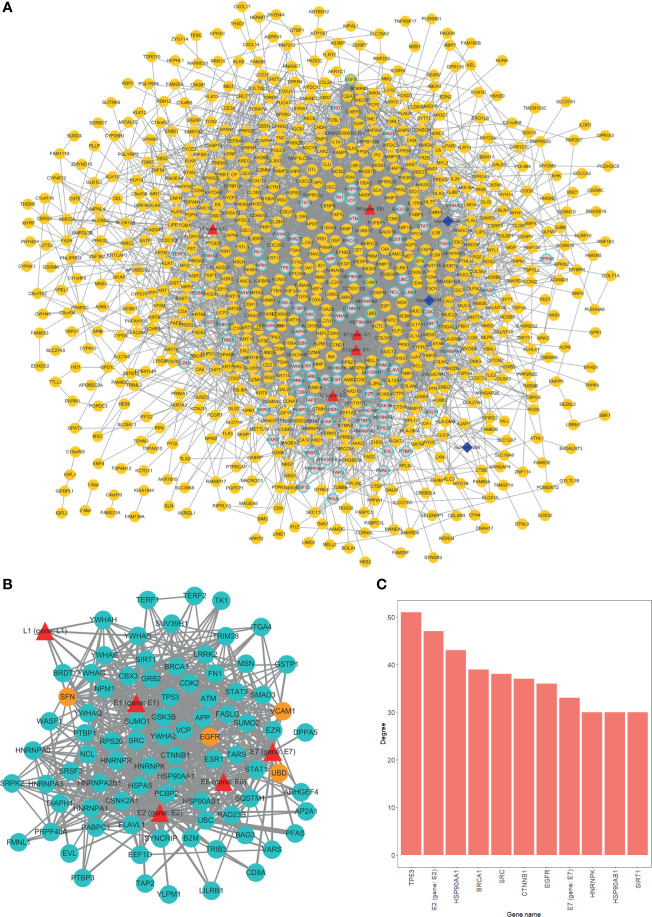
Overview of interaction network. **(A)** Global network of miRNA-mRNA-protein interactions. HPV proteins are marked as red triangles; miRNAs are represented as deep blue diamonds; differentially expressed genes (DEGs) are orange circle; nondifferentially expressed genes are in pink. Circles with light blue border are HPV directly interacted human genes. **(B)** HPV proteins directly regulated subnetwork. Turquoise circles are HPV directly manipulated nondifferentially expressed genes; red triangles are HPV proteins; orange circles are HPV directly manipulated DEGs. **(C)** Top 11 genes degree distributions of HPV proteins directly regulated subnetwork.

With virus protein-human protein, mRNA-mRNA (differentially expressed), and miRNA-mRNA interaction pairs, we constructed a miRNA-mRNA-protein interaction network. The result showed that a considerable number of human genes are regulated by HPV proteins. Thus, the genes that are modulated by HPV viral protein may possibly be the key factors that induce tumor occurrence (**Figure 3A**). Overall, most of the genes directly regulated by HPV are not differentially expressed gene. This suggests that HPV-regulated genes have similar expression pattern in HPV− tumors.

We further extracted a subnetwork that contains only HPV protein and its regulated genes to investigate the manipulation details. From the subnetwork, four genes were differentially expressed and are listed as follows, EGFR, SNF (SWI/SNF related member), ubiquitin D (UBD), and vascular cell adhesion molecule 1 (VCAM1) (**Figure 3B**). Further findings indicated that the variation between HPV+ cancer and HPV− cancer can possibly be attributed to the effect of HPV regulation of those four genes. Through degree analysis of subnetwork, we showed that degrees of tumor suppressor gene TP53 (regulated by HPV E7 protein) are the highest of all genes (**Figure 3C**). This result indicates that the regulation of TP53 by HPV is a crucial mechanism for HPV+ tumor to occur.

### 2.4 EGFR Is the Crucial Gene That Regulate HPV+ Tumor Differentially Expressed Genes

**Figure 4:**
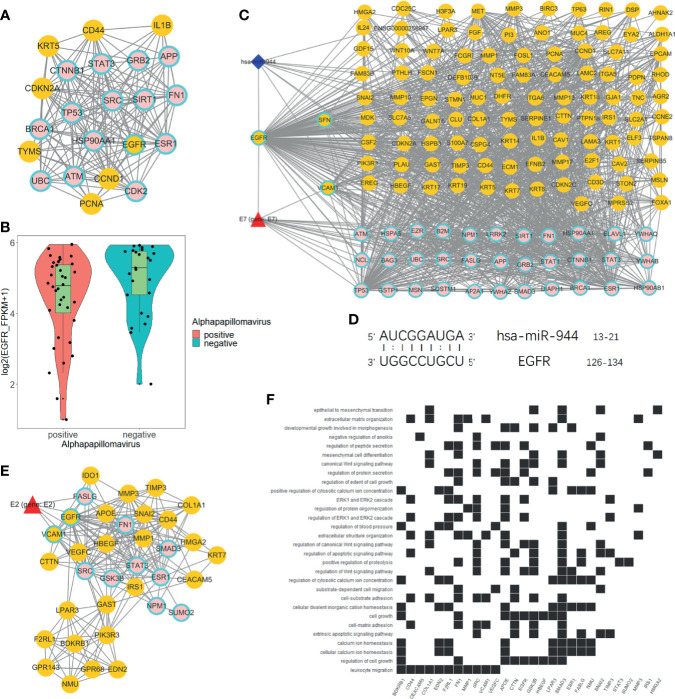
Analysis of EGFR-related network. **(A)** Hub genes of global network, including 15 HPV manipulated genes; seven DEGs are not regulated by HPV and one HPV regulated DEG. **(B)** EGFR express status in HPV+ and HPV− samples respectively according to RNA-Seq FPKM from ICGC PCAWG project CESC and HNSC data. **(C)** Subnetwork of EGFR first neighbors. **(D)** EGFR RNA binding prediction site with hsa-miR-944 using RIsearch2 software. **(E)** MCODE-calculated EGFR containing network cluster. **(F)** EGFR containing cluster GO and KEGG enrichment analysis.

We used degree = 55 to screen hub nodes of miRNA-mRNA-protein network, and 22 nodes were selected. Out of the 22 genes, eight genes are DEGs. Whereas 15 genes are direct HPV-regulated genes and nodes like TP53, BRCA1, EGFR, and CTNNB1 are classic tumor-related genes (**Figure 4A**). Remarkably, EGFR is the only hub node that belongs to both DEGs and directly interacts with HPV (**Figure 4B**). We further extracted a subnetwork constructed with EGFR and its first neighbor. The result showed that EGFR interacts with a considerable amount of DEGs, and it is also regulated by hsa-miR-944 and HPV protein E7 (**Figure 4C**). This indicates that EGFR is an essential gene that regulates the differentially expressed genes.

Furthermore, we predicted that EGFR is regulated by hsa-miR-944 and that the upregulation of has-miR-944 caused the downregulation of EGFR (**Figures 1J**, **4B**). In order to confirm whether hsa-miR-944 combine stably with EGFR, RIsearch2 software was used for RNA combined analysis. The result shows that EGFR and hsa-miR-944 have low-energy binding site at 3′ end of hsa-miR-944 (**Figure 4D**). Our findings revealed that EGFR is regulated by both E7 protein and hsa-miR-944. This shows that E7 protein does not only induces carcinogenesis in HPV+ tissues but also causes the difference in appearance in HPV+ and HPV− tumor.

A module with EGFR was identified using module analysis of global network. Since gene in the same module interacts closely, there is a possibility that they can participate in the same biological process. HPV protein E2, a key protein that plays a pivotal role in HPV infection from the early stage to the late stage, was also identified (**Figure 4E**). This finding suggests that EGFR participates in all HPV infection stages and could probably influence tumor development and prognosis. Likewise, GO and KEGG enrichment analyses revealed that the module genes are enriched with EGFR downstream pathways and participates in several functions, including development regulation, epithelial regulation, and mitogen-activated protein kinase (MAPK) pathway (**Figure 4F**). This implies that EGFR downstream pathways are regulated by E2, and it can influence oncogenesis at late stage of infection.

### 2.5 Activation of EGFR-Related Pathway Is an Important Factor That Decreases Survival

**Figure 5:**
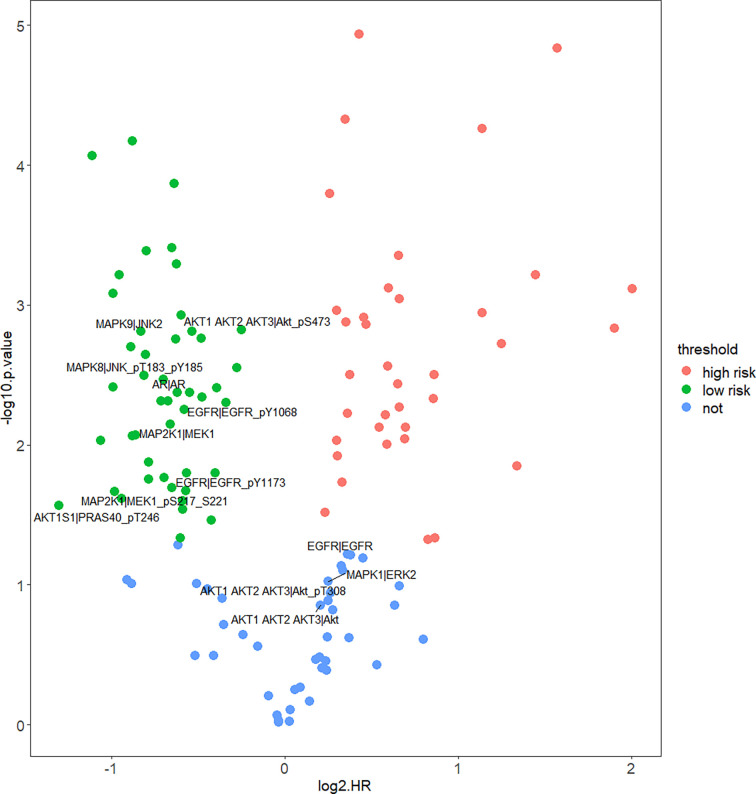
Correlation of protein expression and HPV+ patients’ survival. Figure shows COX proportional hazard regression. It measured correlation significance of protein expression and HPV+ patients’ survival. Only differentially expressed proteins (DEPs) of HPV+ *vs.* HPV− are displayed. Proteins which *p* < 0.05 are significantly correlated with HPV+ patients’ survival, log2HR >0 is positively correlated with survival, log2HR <0 is negatively correlated with survival.

In order to figure out how EGFR influence prognosis, we merged reverse-phase protein array (RPPA) data of CESC and HNSC and a total of 133 proteins were obtained. Student’s *t*-test was used to test for the differences between HPV+ and HPV− groups. Interestingly, 123 proteins were differentially expressed and only 10 proteins were not significant. Yet, TP53 was not differentially expressed at the protein level. Subsequently, COX proportional hazard regression model was used to evaluate the correlation between survival time and deferentially expressed proteins in HPV+ tumor patients. The results suggest that the expressed level of EGFR did not have any significant relationship with prognosis. Notably, there was a negative correlation between all tyrosine residue phosphorylated forms of EGFR and patients’ survival. Phosphorylation on site pY1068 and pY1173 representing EGFR was activated to form a dimer that binds with its ligand thus, further activating downstream pathways like PI3K/Akt, MAPK, and WNT pathway. We also highlighted that amphiregulin (AR), an EGFR ligand, and some significant proteins belong to PI3K/Akt or MAPK pathway. Those downstream proteins also showed similar properties of phosphorylated form of EGFR that are significantly negatively correlated with survival (**Figure 5**).

### 2.6 EGFR Modulates Cancer Prognosis Through the Regulation of Immune and DNA Repair Pathway

**Figure 6:**
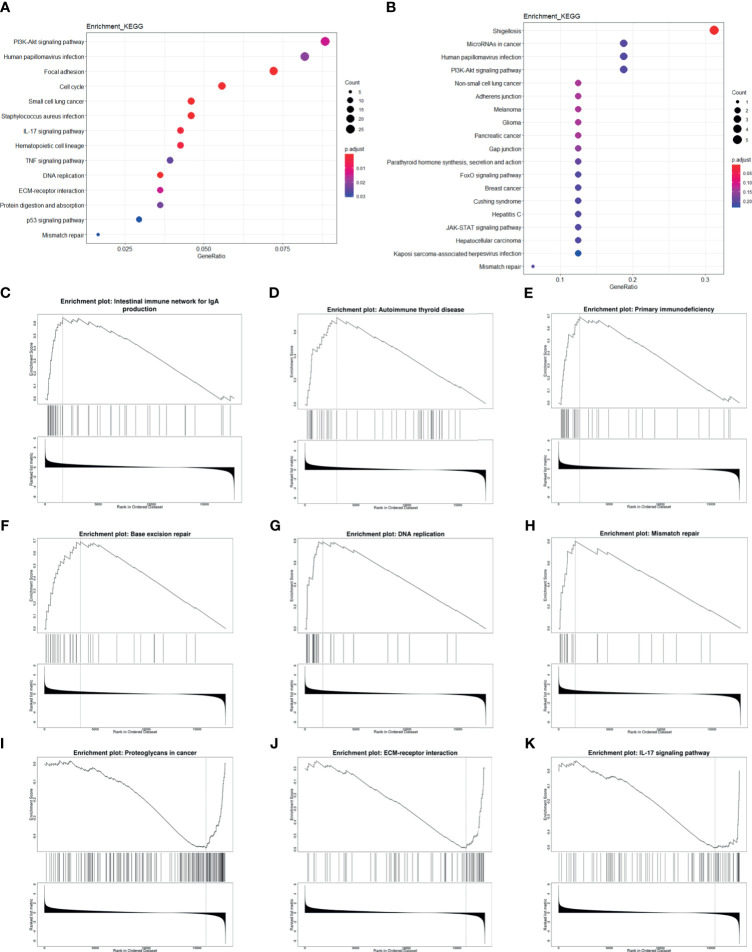
Enrichment analysis of mRNA and miRNA targets. **(A, B)** Top KEGG-enriched terms of differentially expressed genes and differentially expressed miRNA target genes, respectively. **(C–K)** GSEA-enriched terms, in which genes were sorted by log2FC.

In order to further find out the prognosis-related pathways, we carried out KEGG enrichment analysis of HPV+ *versus* HPV− DEGs and mRNA targets of differentially expressed miRNAs (DEmiRs). The top enriched term list showed “human papillomavirus infection,” demonstrating that HPV activates a unique pathway different from HPV− cancer. For DEG enrichment analysis, immune-related pathway (such as IL-17 signaling pathway and TNF signaling pathway), cancer-related pathway, and DNA-related pathway were shown, and it could be correlated with prognosis. Remarkably, EGFR-related pathways, “PI3K/Akt pathway” and “ECM-receptor interaction,” were also included in the top list (**Figure 6A**). For DEmiR target enrichments, numerous cancer-related pathways were shown, and EGFR-related pathway, “PI3K/Akt pathway”, was also enriched (**Figure 6B**). These results suggest that HPV+ cancer shows different prognosis-related pathway activation compared with HPV− cancer, even in cancer-related pathway. Gene Set Enrichment Analysis (GSEA) further showed that immune-related pathways and tumor-related pathways were inactivated and DNA repair pathways were activated in HPV+ cancer groups compared with HPV− groups. All these activated and inactivated pathways can possibly enhance better prognosis of HPV+ cancer (**Figures 6C–K**). Since EGFR is the hub gene of DEG network, EGFR can possibly affect the prognosis of tumors through the regulation of immune, tumor, and DNA repair pathway.

## 3 Discussion

HPV is a tissue-specific oncogenic virus that specifically infects epithelium tissues. The mechanisms of HPV-induced squamous cell carcinoma are probably not similar to the mechanisms of HPV nonassociated squamous cell carcinoma. Since both mechanisms cause tumorigenesis, it suggests that their gene expression patterns are somewhat common. Based on this hypothesis, our study compared HPV+ and HPV− tumors at multiomics level in order to identify similar and different underlining molecular mechanisms. Although HPV can infect various types of epidermal tissues, CESC and HNSC are the most common types of HPV-induced squamous cell carcinoma. Considering the representative data and limited data volume, we combined both CESC and HNSC data into our study. Also, since our study focuses on HPV-induced tumorigenesis rather than ontogenesis, we merged the two cancer data for analysis instead of separate analysis. All HNSC samples included in this study were oropharynx carcinomas, which avoids the influence of non-HPV-associated HNSCs on the final results.

Recent reports revealed that HPV+ patients show better prognosis than HPV− patients in certain types of tumor (23). In addition, our study is consistent with this result despite merging data from two different types of cancer. It implies that the better prognosis of HPV+ tumor is consistent across different tumor types. This effect is probably related to the difference in the expression pattern of prognosis-associated genes. Although, different in mechanisms, both HPV− and HPV+ tumors express similar pattern in tumor-associated genes. Virus infection influence host gene expression pattern mainly through direct regulation by virus-derived proteins, and this process rarely induce gene mutations (24). The tumors that are not driven by virus usually show mutations at oncogenes and/or tumor suppressor genes. In the total of 84 HPV-regulated genes, only four genes (EGFR, SNF, UBD, and VCAM1) were differentially expressed when compared with HPV− tumor. Among the four genes, SNF and VCAM1 directly interact with EGFR through string estimation; this suggests that HPV-regulated DEG tends to interact, and that they participate in similar biological processes that affect patient’s survival.

Our RNA-Seq PCA showed that HPV+ tumors are distributed in different areas against HPV− tumor at the first principal component. Although the dispersion within group is not obvious, we believe that HPV infection status probably influences gene expression more than the primary tumor site. The 834 DEGs obtained from differentially expressed analysis further confirm that gene expression patterns of HPV+ tumors are different from HPV− tumors. Although the clustering analysis showed that the clustering of samples was basically the same as that of HPV infection, a small number of HPV+ and HPV− clustered together. There are two possible reasons for this phenomenon. First, the HPV expression level of the samples may be very low, which may induce their gene expression patterns closer to that of HPV− tumors. Second, these samples were probably infected by low-risk HPV that rarely induce cancer, which implies that their oncogenesis ability is relatively lower. In spite of diversity in mRNA expression, miRNA expression between HPV+ and HPV− shows little differences. However, based on the enrichment analysis of DEmiRs target genes, we showed that there are several differences in tumor-related pathways of HPV+ and HPV− cancers. One possible reason for this is that only a small percentage of miRNAs are differentially expressed in HPV+ and HPV− tumors. In addition, these differentially expressed miRNAs are highly correlated with tumor-related biological processes.

Since certain mutations occur across different cancer types (25), we therefore focus on differences in mutation types between HPV+ and HPV− tumors. Our study showed that TP53 mutation rate in HPV+ tumor is dramatically lower than in HPV− tumor. TP53 is an important tumor suppressor gene. The mutation of tumor suppressor gene is considered more serious than its dysfunction. HPV+ cancer patients showed better outcome than HPV− cancer patients and that can probably be attributed to low TP53 mutation rate (7, 26). We also showed that genes that belong to the same family or participate in the same pathway have higher mutation rate. For instance, mucin glycoprotein-encoded genes MUC16, MUC17, and MUC4 ranked among the top 30 mutated genes. This suggests that mucin glycoprotein mutation is a signature of HNSC and CESC (27). Moreover, MUC4 mutation rate in HPV+ samples are obviously higher compared with HPV− samples. We believe that mucin glycoprotein subtype and their mutation rates could be a latent biomarker for tumor classification.

Virus protein expression and regulation of biological function are usually diverse in HPV infection stage. For HPV, functional proteins like E1, E2, E5, E6, and E7 are highly expressed at early infection stage. Using these early stage proteins, HPV can hijack DNA and protein synthesis machinery of the cell for self-proliferation. At the final stage, HPV capsid proteins, L1 and L2, are expressed for virus assembly and escape from the cell (28). Reports suggest that, E2, an early-stage protein, possibly participates in the late stage of HPV replication by activating DNA damage response (29). Our result shows that, EGFR is regulated by HPV oncoprotein E7 and that it takes part in E2-related regulation unit. This suggests that EGFR is involved in E2-regulated DNA damage response (30). Also, DNA-related functions of HPV+ cancers are significantly activated compared with that of HPV− cancers. Beside HPV proteins, differentially expressed miRNAs also participate in EGFR regulation. Although differentially expressed miRNAs and their targets are rare, it is not accidental that differentially expressed miRNA targets EGFR (hypergeometric test *p* < 0.001).

Since EGFR is a potent oncogene, EGFR dysregulation will cause several forms of cancer. High proportion of nonsmall-cell lung carcinomas expresses EGFR and the EGFR mutant as its signature (31). Likewise, EGFR has become a biomarker of HNSC (15). In our study, EGFR shows different express pattern for HPV+ and HPV− cancers. EGFR is a critical receptor that transduces epithelium growth and developmental signal into the cells. It plays an important role in epithelial stem cell division and differentiation. HPV does not only infect the basal cells of epithelium tissues but it also requires epithelial development for its replication. Hence, regulation of basal cell differentiation is a crucial control strategy for HPV replication. On the other hand, HPV− cancers do not require epithelium differentiation for its replication. Therefore, EGFR downregulation is a possible potential strategy that targets HPV-specific lifecycle. Tyrosine-phosphorylated EGFR is the activated form of EGFR. Only the activated form of EGFR can serve as a receptor and receive extracellular signals. Downregulation of the overall expressed EGFR can possibly decrease the expression of phosphorylated EGFR, thus inhibiting the downstream signaling pathway of EGFR (32). It has been reported that continuous hyperfractionated accelerated radiotherapy is more effective for HNSCs with high EGFR expression than HNSCs with low EGFR expression (33). Considering that HPV+ cancer induces higher EGFR express level upon study provides us a potential specific HPV+ cancer therapeutic method.

Although, there are limitations to this study. We only considered the effect of HPV infection on tumors but ignored the potential effect of different subtypes of HPV (HPV-16, HPV-33, HPV-18) on gene expression. Whether EGFR expression level is higher in some HPV subtypes or lower in other subtypes during infection still need to be explored.

In summary, HPV+ cancer is significantly different from HPV- cancer in many aspects like DNA mutation, mRNA and protein expression. The initiation of cancer in HPV+ cells results from the regulation of biological processes related to host development by viral proteins. In contrasts, HPV− cancer is activated by several categories of risk factors and is highly related to TP53 mutation. Although distinct in mechanisms, both HPV+ and HPV− cancers are triggered by onco-related gene dysregulation. This study showed that EGFR is possibly the core molecule that affects immune and cancer-related biological processes and it can eventually cause prognosis differences between HPV+ and HPV− cancers.

## 4 Materials and Methods

### 4.1 Data Source

CESC and HNSC RNA-Seq read counts data of the PCAWG project were obtained from the ICGC database (https://icgc.org/). In the PCAWG project, information on HPV infection in each of the samples was from a study recently published by Zapatka et al. (34). CaPSID, P-DIP, and SEPATH pipelines were used for detection of HPV reads from PCAWG samples in the study by Zapatka, and samples with HPV reads that were detected in at least two pipelines were considered infected. After screening, 20 CESC samples (19 HPV+, one HPV−) and 41 HNSC samples (17 HPV+, 24 HPV−) were included in this study. Simple nucleotide variation (SNV) data, including 305 CESC samples and 510 HNSC samples, are from Genomic Data Commons Data portal (GDC, http://portal.gdc.cancer.gov/). The source of reverse-phase protein array (RPPA) level 3 data and clinical data of 173 CESC samples and 212 HNSC samples were obtained from the FireBrowse database (http://firebrowse.org/).

### 4.2 Principal Component Analysis

PCA was applied for data dimension reduction. Sample distribution confidence intervals of each sample groups were displayed. The samples located far away from confidence interval ellipse were considered outliers and were deleted in the subsequent exploration.

### 4.3 Differentially Expressed Analysis

Limma package of R was used for RNA-Seq data and miRNA-Seq data differentially express analysis. DEGs and DEmiRs were identified by *p. adjust.* <0.05 and |log2FC| >1.2. BH method was applied for *p-*value adjustment. Student’s *t*-test was used to analyze differentially expressed RPPA data. Proteins with *p-value <*0.05 were regarded as differentially expressed proteins (DEPs).

### 4.4 Survival Analysis

Clinical data from FireBrowse database were used for the prediction in survival differences of the two groups. To compare the data from the HPV+ tumor patients and HPV− tumor patients, Kaplan-Meier method was used for survival rate prediction and Kaplan-Meier plot (KM plot) was used to determine the survival curve. COX proportional hazard regression model was used to predict the correlation between differentially expressed protein (from RPRA data) and HPV+ patients’ survival time. *p* < 0.05 was considered to significantly correlate with survival time, HR >1 was considered positive correlation with survival time, and HR <1 was considered to be negative correlation with survival time.

### 4.5 miRNA Target Prediction

TargetScan (http://www.targetscan.org/vert_72/) and miRDB (http://mirdb.org/) database were used for differentially expressed miRNA target prediction. Targets that appeared in both databases were considered well predicted. RIsearch2 software was used for further verification of specific interesting miRNA-mRNA interaction.

### 4.6 SNV Analysis

Single nucleotide variation (SNV) data of CESC and HNSC project were downloaded from the TCGA GDC data portal. The processed MAF data used in this study was downloaded from MUSE software processed. Maftools R package was used for MAF file data mining. Maftools was likewise used for statistical analysis of gene mutation and data visualization.

### 4.7 HPV-miRNA-mRNA Network Construction and Analysis

HPV-human protein interaction prediction data were downloaded from P-HIPSTer (http://phipster.org/), a database that predicts virus-human protein interactions based on structural information. Differentially expressed mRNA interactions were predicted by String database (https://string-db.org/). MCODE plugin of Cytoscape (version 3.7.0) was used for network module identification. The parameters for MCODE were set as default. The degree of calculation was determined by NetworkAnalyzer, and genes with degree higher than the threshold were defined as hub genes.

### 4.8 Enrichment Analysis

KEGG pathway enrichment analysis was determined using R package clusterProfiler. Hypergeometric test was used for terms significance testing. The *p. adjust.* <0.2 was set as the threshold of significantly enriched terms. Webtools WEBGESTALT (http://www.webgestalt.org/) was used for GSEA enrichment analysis and result visualization (35).

## Data Availability Statement

The original contributions presented in the study are included in the article/supplementary material. Further inquiries can be directed to the corresponding authors.

## Author Contributions

JQ, FH, and YW conceived the study. JQ, FH, and YW designed the experiment. JQ performed the experiment and computational analysis. YG, ZD, HN, and YC collected the data. JQ wrote the manuscript. OO, WZ, YQ, and LL provided valuable suggestions to improve the manuscript. TS and ZZ provided the professional consulting support. All authors contributed to the article and approved the submitted version.

## Funding

This work was supported by the National Natural Science Foundation of China grants to YW (81772188) and ZZ (81571999 and 81871652); Natural Science Foundation of Heilongjiang Province grant to LL (LH2019H004); and Heilongjiang Postdoctoral Scientific Research Developmental Fund to LL (LBH-Q19146).

## Conflict of Interest

The authors declare that the research was conducted in the absence of any commercial or financial relationships that could be construed as a potential conflict of interest.

## Publisher’s Note

All claims expressed in this article are solely those of the authors and do not necessarily represent those of their affiliated organizations, or those of the publisher, the editors and the reviewers. Any product that may be evaluated in this article, or claim that may be made by its manufacturer, is not guaranteed or endorsed by the publisher.
